# Expression Analysis of SOX14 during Retinoic Acid Induced Neural Differentiation of Embryonal Carcinoma Cells and Assessment of the Effect of Its Ectopic Expression on SOXB Members in HeLa Cells

**DOI:** 10.1371/journal.pone.0091852

**Published:** 2014-03-17

**Authors:** Jelena Popovic, Danijela Stanisavljevic, Marija Schwirtlich, Andrijana Klajn, Jelena Marjanovic, Milena Stevanovic

**Affiliations:** Laboratory for Human Molecular Genetics, Institute of Molecular Genetics and Genetic Engineering, University of Belgrade, Belgrade, Serbia; Baylor College of Medicine, United States of America

## Abstract

SOX14 is a member of the SOXB2 subgroup of transcription factors implicated in neural development. Although the first *SOX14* gene in vertebrates was cloned and characterized more than a decade ago and its expression profile during development was revealed in various animal model systems, the role of this gene during neural development is largely unknown. In the present study we analyzed the expression of SOX14 in human NT2/D1 and mouse P19 pluripotent embryonal carcinoma cells. We demonstrated that it is expressed in both cell lines and upregulated during retinoic acid induced neural differentiation. We showed that SOX14 was expressed in both neuronal and non-neuronal differentiated derivatives, as revealed by immunocytochemistry. Since it was previously proposed that increased SOXB2 proteins level interfere with the activity of SOXB1 counteracting partners, we compared expression patterns of SOXB members during retinoic acid induction of embryonal carcinoma cells. We revealed that upregulation of SOX14 expression is accompanied by alterations in the expression patterns of SOXB1 members. In order to analyze the potential cross-talk between them, we generated SOX14 expression construct. The ectopic expression of *SOX14* was demonstrated at the mRNA level in NT2/D1, P19 and HeLa cells, while an increased level of SOX14 protein was detected in HeLa cells only. By transient transfection experiments in HeLa cells we showed for the first time that ectopic expression of SOX14 repressed SOX1 expression, whereas no significant effect on SOX2, SOX3 and SOX21 was observed. Data presented here provide an insight into SOX14 expression during *in vitro* neural differentiation of embryonal carcinoma cells and demonstrate the effect of its ectopic expression on protein levels of SOXB members in HeLa cells. Obtained results contribute to better understanding the role of one of the most conserved SOX proteins.

## Introduction

Members of the *SOX* gene family code for transcription factors that either activate or repress transcription of target genes which participate in important biological processes during embryonic development [1]. Based on HMG box homology and intron-exon structure, *SOX*/*Sox* genes are divided into 10 distinct groups, designated from A to J [2]. *SOXB*/*SoxB* group members (*Sox1, Sox2, Sox3, Sox14* and *Sox21*) are of particular interest since they play a major role in neural development. They participate in the earliest events of central nervous system (CNS) differentiation in *Drosophila, Xenopus*, chicken and mouse embryos [3]–[7]. Based on sequence analysis and functional studies in vertebrates, it was proposed that *SOXB*/*SoxB* genes can be further divided into subgroup SOXB1, comprising activators (*SOX1*/*Sox1, SOX2/Sox2* and *SOX3*/*Sox3*) and subgroup SOXB2, consisting of repressors (*SOX14/Sox14* and *SOX21*/*Sox21*) [7]. SOXB1 transcription factors show functional similarity in the regulation of the neuronal phenotype [3]. Comparative analyses of the expression patterns of *SoxB1* genes in chicken [7]–[9] and mouse embryos [3], [10]–[11] have indicated that the expression of these genes is strongly correlated with the development of neural primordial tissues, starting from the neural plate stage and continuing to the ventricular zone of the later CNS [12]–[16]. SOXB2 transcription factors are also expressed in the CNS and it was postulated that they have roles in the specification of a particular subset of neurons, rather than neural development in general [17].

The expression pattern of the *Sox21* gene correlates with the expression of *SoxB1* genes in the neural primordia, whereas *Sox14* is expressed in the limited domains of the post-primordial neural tissues [7]. During the early stages of CNS development, it was proposed that vertebrate SOXB2 transcription factors target the same genes as SOXB1 activators, but with the opposite effect [7]. Thus, it was postulated that regulation of target gene expression is probably the result of a fine counterbalance between SOXB1 and SOXB2 activities. It was suggested that an increase in SOXB2 protein levels activates proneural proteins, which subsequently interfere with SOXB1 function, leading to differentiation of a neural progenitor towards neuronal phenotype [18].

The *SOX14/Sox14* gene has been identified in many vertebrate species, including human, mouse, chicken, platypus and fish [7], [19]–[24]. Comparative sequence analysis has revealed remarkable identity among SOX14 orthologues, suggesting that it is one of the most conserved SOX proteins during evolution [25]. A high level of conservation indicates that the SOX14 protein has been under strong evolution pressure, during which it has retained its functional properties [25]. To date, no *SOX14/Sox14* mutations associated with human genetic disorders or animal phenotypes have been described. The evolutionary conservation and lack of any known mutated phenotype suggest that *SOX14*/*Sox14* might have an essential role during development and that loss of its function might lead to a lethal phenotype.

There is a limited number of studies in various model systems, mostly focused on *Sox14* expression during neural development, which have proved that its expression is very narrow, compared to the expression of other members of the SOXB subgroup [7]. *Sox14* gene expression analysis during mouse and chick development has shown that its expression pattern is restricted to a limited population of neurons in the developing brain and spinal cord [4]. In the spinal cord, *Sox14* is expressed in a subset of interneurons in a defined dorsoventral position adjacent to ventral motor neurons and it has been suggested that it is involved in the specification of this group of interneurons [4]. Expression analysis in *Xenopus* revealed that *sox14* expression is restricted to the hypothalamus, dorsal thalamus and the optic tectum [21]. Recent work by Delogu et al. has revealed that *Sox14* is expressed in a subset of GABAergic neurons in mouse diencephalon [26]. It has been shown that its expression is required for proper distribution of neurons among different nuclei of the subcortical visual shell and for development of a functional network supporting light-entrained circadian behaviour [26].

Although the human *SOX14* gene was first cloned and characterized more than a decade ago [19], [23], followed by identification of *Sox14* genes in numerous organisms, it is still the least examined member of the SOXB subgroup. Previously, we have cloned and characterized human *SOX14* gene and determined its promoter and regulatory elements involved in transcriptional regulation of its expression. We have also identified transcription factors NF-Y and Foxa2 as positive regulators of *SOX14* expression and proposed that the Sonic hedgehog signaling pathway involved in up-regulation of *SOX14* expression might be, at least in part, mediated by FOXA2 [27]–[28].

The aim of this study was to analyze *SOX14/Sox14* expression during retinoic acid (RA) induced neural differentiation of pluripotent human NT2/D1 and mouse P19 embryonal carcinoma (EC) stem cells, which display properties similar to embryonic stem cells [29]–[31]. Terminally differentiated NT2/D1 and P19 neurons (NT2-N and P19-N, respectively) exhibit properties of post-mitotic polarized cells that express neurofilaments, generate action potentials and calcium spikes, express, release, and respond to neurotransmitters, and form functional synapses [30], [32]–[34]. Accordingly, these cell lines provide valuable *in vitro* model systems for studying molecular mechanisms underlying human and mouse neural differentiation. Further, we wanted to study the effect of ectopic SOX14 expression on the activity of SOX-responsive reporter gene and to analyze whether its overexpression interferes with expression of SOXB1 transcription factors *in vitro*.

The results presented here contribute to better understanding the role of one of the most conserved SOX proteins.

## Materials and Methods

### Cell culture and differentiation

Human NT2/D1 EC stem cells (ATCC^®^ CRL-1973™) were maintained in Dulbecco's Modified Eagle's medium (DMEM) supplemented with 10% fetal bovine serum (FBS), 4500 mg/L glucose, 2 mmol/L L-glutamine and penicillin/streptomycine (all from Invitrogen™, NY, USA), at 37°C in 10% CO2 as previously described [29]. Cells were induced to differentiate in culture by addition of 10 μmol/L all-trans retinoic acid (RA; Sigma-Aldrich, MO, USA) for 4 weeks. A neuron-enriched population was isolated in accordance with Pleasure et al. [34]. Briefly, following RA induction, cells were replated at lower density (1∶6). After 2 days in culture, neuron-like cells were detached by tapping mechanically on the side of the tissue culture plate and re-plated on Matrigel™ (Becton Dickerson, NJ, USA) coated dishes. Cells were grown for the following 10 days in the presence of mitotic inhibitors: 1 mmol/L cytosine arabinoside, 10 mmol/L uridine and 10 mmol/L 5-fluoro-5-deoxyuridine (all from Sigma-Aldrich).

Mouse P19 EC stem cell line (ATCC^®^ CRL-1825™) [30] was grown in DMEM containing 10% FBS, 4500 mg/L glucose, 2 mmol/L L-glutamine and penicillin/streptomycine (all from Invitrogen™), at 37°C in 5% CO2. Neural differentiation was induced by RA as described by Rudnicki et al. and McBurney with slight modifications [31], [35]. Briefly, cells were plated into bacterial-grade petri dishes in the growing medium, supplemented with a final concentration of 1 μmol/L RA. After a five-day induction period, aggregates were gently plated into Matrigel-coated tissue culture dishes and incubated for 7 days in DMEM/F12 (Invitrogen™) medium supplemented with 5% FBS. During the induction period, medium supplemented with fresh RA was replaced every 48 hours in NT2/D1 and P19 cultures.

HeLa (ATCC CCL-2) cells were maintained in DMEM supplemented with 10% FBS and 1% non-essential amino acids (NEAA; Invitrogen) at 37°C in 5% CO_2_.

### Western blot

To obtain whole cell lysates, cells were briefly rinsed with ice-cold PBS and extracted in ice-cold lysis buffer containing 1% Triton X-100, 50 mmol/L Tris-HCl (pH 7.5), 250 mmol/L NaCl, 5 mmol/L EDTA and protease inhibitor cocktail (Roche Diagnostics GmbH, Germany). Proteins were quantified by Bradford protein assay (Bio-Rad Laboratories, Inc., CA, USA). Samples were separated by SDS-PAGE on 10% or 12% resolving gels and then electrotransferred to Immobilon-P Transfer Membrane (Millipore, MA, USA). After blocking with 5% non-fat milk at room temperature (RT) for 1 h, membranes were incubated for 1 h at RT with the following primary antibodies: rabbit polyclonal antibodies against SOX14 (Abcam, Cambridge, UK, ab149047, diluted 1∶400), mouse monoclonal anti β-III tubulin (T-8660, Sigma-Aldrich, diluted 1∶10000), rabbit polyclonal GFAP (DakoCytomation, Glostrup, Denmark, Z 0334, diluted 1∶20000), mouse monoclonal anti α-Tubulin (Calbiochem, MA, USA, CP06, diluted 1∶30000), mouse monoclonal antibody against GAPDH (Abcam, ab9484, diluted 1∶5000), rabbit monoclonal antibody against SOX1 (Abcam, ab109290, diluted 1∶1000), mouse monoclonal antibody against SOX2 (R&D, MAB2018, diluted 1∶2500) or rabbit polyclonal antibody against SOX2 (Active Motif, 39824, diluted 1∶2500), rabbit polyclonal antibody against SOX3 (Abcam, ab42471, diluted 1∶2000), mouse monoclonal antibody against SOX21 (Abcam, ab56837, diluted 1∶500), mouse monoclonal antibody against SNAP25 (Sternberger Monoclonals, diluted 1∶1 L) and rabbit monoclonal antibody against OCT4 (Cell Signaling, #2840, diluted 1∶1000). Afterwards, the membranes were incubated for 1 h at RT with the following secondary antibodies: horseradish peroxidase-conjugated anti-mouse and anti-rabbit IgG (Amersham Biosciences, NJ, USA, diluted 1∶10000). Immunoreactive bands were detected by chemiluminescence (Immobilion substrate, Millipore, MA, USA).

### Immunocytochemistry

After plating on cover slips, cells were fixed in 4% paraformaldehyde (PFA) for 20 min at RT. Cells were permeabilized in 0.1% Triton X-100 and blocked in 5% bovine serum albumin (BSA), 0.1% Triton X-100 or 10% normal goat serum in PBS for 1 h at RT. Primary antibodies were diluted in PBS containing 5% BSA, 0.1% Triton X-100 or 1% BSA, 0.05% Tween^®^ 20 (Sigma-Aldrich) and incubated overnight at 4°C as follows: rabbit polyclonal anti-SOX14 (Abcam, ab149047, diluted 1∶200), mouse anti-MAP2 (Abcam, ab11267, diluted 1∶500), rabbit polyclonal anti-GFAP, (DakoCytomation, Z 0334, diluted 1∶2000) and mouse anti α-Tubulin (Calbiochem, CP06, diluted 1∶200). Cover slips were washed 3×10 min in 0.1% Triton X-100 or 0.05% Tween^®^ 20, prepared in PBS (PBTr and PBT, respectively) and incubated with anti-rabbit or anti-mouse guinea-pig secondary antibodies conjugated either with Alexa Fluor® 594, Alexa Fluor® 488 (Invitrogen™, diluted 1∶500 in 1% BSA-PBT) or DyLight™ 649 for 60 minutes at RT. The anti-GFAP antibody was first labelled with biotinylated goat anti-rabbit IgG (Vector, Burlingame, CA, USA) for 1 h at RT in 1% BSA, followed by Cy3-streptavidin (Jackson ImmunoResearch, West Grove, PA, USA, diluted 1∶5000) diluted in PBS for 1 h at RT. Nuclei were stained with 0.1 mg/ml diamino phenylindole (DAPI; Sigma-Aldrich). Samples were viewed under an Olympus IMT-2 and images were taken using a digital camera (Olympus C-5050), or by a Leica TCS SP8 confocal microscope and Leica Microsystems LAS AF-TCS SP8 software (Leica Microsystems).

### Generation of expression constructs

The complete *SOX14* coding sequence was amplified by PCR from genomic clone SOX14P32.2*Xba*I [19], using primers 5′-CTCGTCTGCAGAACCCTTGCAC-3′ (forward) and 5′-GACCCCGGAGGCGTCTGCAG-3′ (reverse). PCR reaction was performed using KAPA 2G Fast HotStart Ready Mix (Kapa Biosystems, MA, USA) according to manufacturer's protocol. The PCR product was eluted from agarose gel and cloned into pJET1.2 vector using a CloneJET* PCR Cloning Kit (Fermentas, Thermo Fisher Scientific, USA). The selected clone was fully sequenced in order to verify that no mutations were introduced by PCR. Using *Bgl*II digestion, the fragment containing the *SOX14* coding region was released from pJET1.2 and then subcloned into pcDNA3.1 vector using *BamH*I compatible ends. The *SOX21* coding region was amplified from genomic DNA using primers 5′-CCAACATTGATTTCCTCCGG-3′ (forward) and 5′- CCTTAAGGCAGCGCTCGTACCTATAC -3′ (reverse) and the PCR product was cloned into pJET1.2 vector. The fragment containing the *SOX21* coding region was released from pJET1.2, and then subcloned into pcDNA3.1 vector using *Xba*I*/Xho*I compatible ends. Full-length human *SOX3* cDNA was released from clone Id 7939708 (Open Biosystems) using *EcoR*I restriction enzyme and cloned into pcDNA3.1 vector.

### Transfection assays

NT2/D1, P19 and HeLa cells were seeded in 6-well plates and grown for 1 day until they reached 90% confluency. Cells were transfected with 3 μg of either pcDNA3.1, pcDNA3.1/SOX14, pcDNA3.1/SOX3 or pcDNA3.1/SOX21 construct using Lipofectamine® 2000 reagent (Invitrogen™, USA) or PEI transfection reagent (Polyethyleneimine “MAX”, Polysciences.Inc, Cat No 24765) according to the manufacturer's protocol. Cells were collected 24, 48 and 72 h after transfection.

For luciferase assay, HeLa cells were seeded at an approximate 90% confluence in 24-well plates. The following day, cells were co-transfected with 300 ng of SOX-responsive reporter construct 3SXluc and 300 ng of either empty pcDNA3.1 or pcDNA3.1/SOX14 using Lipofectamine™ 2000 reagent, according to the manufacturer's protocol. 50 ng of pRLSV40 plasmid (Promega, USA) was used for normalization of transfection efficiency. The luciferase reporter 3SXluc contains 3 SOX consensus binding sites cloned into pTATA luc, which carried the luciferase gene under the control of the beta-globin minimal promoter [36]. Cells were harvested and lysed in Reporter Lysis Buffer (Promega, USA) 24 h after transfection and extracts were assayed for luciferase activity using a Dual-luciferase® Reporter Assay System (Promega, USA).

### RT-PCR analysis

Total RNA was isolated using TRI-Reagent (Ambion®, Invitrogen,USA) according to the manufacturer's instructions. RNA was treated with DNase I using a DNA-Free™ kit (Ambion, Invitrogen) and subjected to cDNA synthesis. Total RNA (1 μg) was reverse transcribed using High Capacity cDNA Reverse Transcription Kit (Applied Biosystems®) according to the manufacturer's protocol. The synthesized cDNAs were used as templates for amplification with primers specific for SOX14 and GAPDH. Primers for SOX14 amplification were as follows: 5′-ATGCACAACTCGGAGATCAGC-3′ (forward) and 5′-ACATACCTGTCCTTCTTGAGC-3′ (reverse). GAPDH was amplified with 5′-GGACCTGACCTGCCGTCTAG-3′ (forward) and 5′-CCACCACCCTGTTGCTGTAG-3′ (reverse) to control for equivalent amounts of cDNA per reaction. Primers used for amplification of mouse actin were as follows: 5′-AGCTGAGAGGGAAATCGTGC-3′ (forward) and 5′-GATGGAGGGGCCGGACTCAT-3′ (reverse). RT-PCRs were performed in 20 μl reactions using KAPA 2G Fast HotStart Ready Mix (Kapa Biosystems,) according to the manufacturer's protocol.

For quantitative PCR analysis, cDNAs were subjected to real time PCR using Power SYBR Green PCR Master Mix (Applied Biosystems®) in 7500 Real Time PCR Systems (Applied Biosystems®). *SOX14* and *GAPDH* cDNAs were amplified using primer sets, as mentioned above. All samples were measured in triplicate and the mean value was considered. The relative level of *SOX14* expression was determined using a comparative quantification algorithm where the resulting ΔΔCt value was incorporated to determine the fold difference in expression (2^−ΔΔCt^). Relative *SOX14* mRNA level was presented as a percentage of mRNA expression in undifferentiated NT2/D1 cells.

## Results and Discussion

### SOX14 expression is upregulated during neural differentiation of EC cells

**Figure 1:**
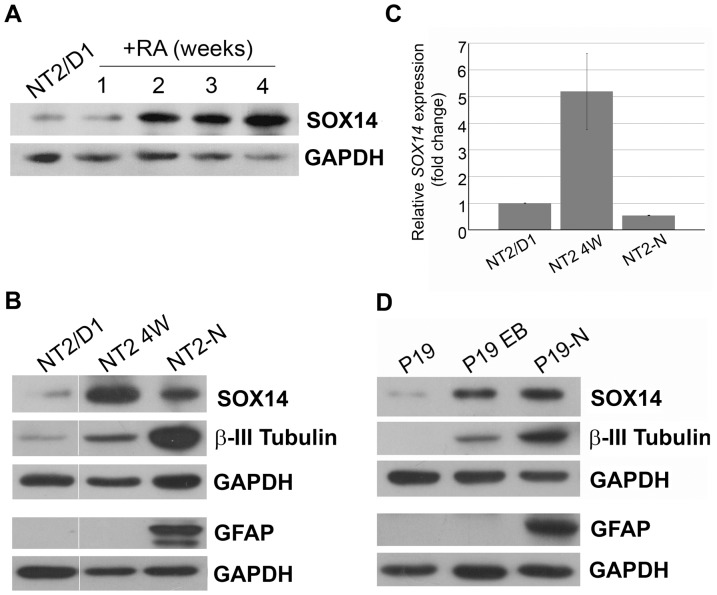
SOX14 expression analysis during RA induced neural differentiation of NT2/D1 and P19 cells. **A**: Western blot analysis of SOX14 expression in undifferentiated NT2/D1 cells treated with RA for 1, 2, 3 and 4 weeks. **B**: Comparison of SOX14 protein level between undifferentiated NT2/D1, cells differentiated for 4 weeks (NT2 4W) and a population of neurons (NT2-N). **C**: qRT-PCR of SOX14 mRNA isolated from NT2/D1, NT2 4W and NT2-N cells. The relative quantities of SOX14 mRNA were calculated as a percentage of the quantity in undifferentiated NT2/D1 cells, which was set as 1. Data are presented as the means ± SD of two independent NT2/D1 differentiation experiments. **D**: Western blot analysis of SOX14 expression in undifferentiated P19 and cells during RA-induced differentiation, including embryoid bodies (P19 EB) and a differentiated neuronal population (P19-N). Progression of neural differentiation was examined by expression analysis of β-III Tubulin and GFAP, as markers of differentiated neurons and astroglial cells, while GAPDH was used as the loading control.

Since SOX14 is considered as a neuronal marker during development, we assumed that RA-induced *in vitro* neural differentiation of human and mouse EC cells could provide an adequate model system for studying *SOX14/Sox14* expression and function. Accordingly, one of our goals was to analyze SOX14 expression during neural differentiation, particularly in terminally differentiated neurons. The progression of neural differentiation was confirmed by expression analysis of β-III Tubulin, the earliest marker of neuronal differentiation [37] and Glial fibrillary acidic protein (GFAP), an intracytoplasmic filamentous protein specific to mature astrocytes [38] (Figure 1B and D).

In order to analyze the human SOX14 expression pattern, we performed Western blot analysis on whole cell lysates obtained from undifferentiated NT2/D1 cells and cells treated with RA for 1, 2, 3 and 4 weeks (Figure 1A). We have shown that SOX14 was expressed at low level in undifferentiated NT2/D1 cells and that its expression was upregulated during neural differentiation, with the maximum level at the final phase of RA induction, after 4 weeks of RA treatment (NT2 4W, Figure 1A).

Further, we compared the level of SOX14 expression between undifferentiated cells, cells treated with RA for 4 weeks and a purified neuronal population (NT2-N) (Figure 1B). Interestingly, the level of SOX14 expression was downregulated in NT2-N compared to NT2 4W (Figure 1B). The reduced expression of *SOX14* in NT2-N was also noticed at the mRNA level. By qRT-PCR we detected an approximate 5-fold increase of the *SOX14* mRNA level in NT2 4W compared to NT2/D1 cells (Figure 1C). On the other hand, *SOX14* mRNA was reduced by approximately 10-fold in the purified NT2-N population compared to NT2 4W, and by approximately 2-fold, compared to undifferentiated NT2/D1 cells (Figure 1C).

Next, the expression of SOX14 was also analyzed in the course of neural differentiation of mouse P19 cells. In the presence of RA, P19 cells form cell aggregates (embryonic bodies - EB) and differentiate into neurons and astrocytes [35]. We performed Western blot analyses on whole cell lysates collected from undifferentiated cells, cell aggregates grown in suspension in the presence of RA for 5 days (final phase of RA induction, designated as P19 EB), and from attached EBs further differentiated for 7 days in culture (designated as P19-N). We have shown that expression of SOX14 was rapidly increased upon RA treatment, with the highest expression level in P19-N (Figure 1D).

Comparison of SOX14 expression in the final phases of RA induction of NT2/D1 and P19 cells (NT2 4W and P19 EB, respectively) and in terminally differentiated neurons (NT2-N and P19-N, respectively) revealed the opposite results. While downregulation of SOX14 expression was observed in NT2-N compared to NT2 4W, its expression was upregulated in the P19-N population, compared to P19 EB. The discrepancy in expression could be due to the variations in protocols used for *in vitro* differentiation of NT2/D1 and P19 cells, or might be caused by the presence of diverse neural derivates in NT2-N and P19-N populations. We speculate that downregulation of SOX14 expression at both the mRNA and protein levels in NT2-N, compared to NT2 4W, might be a consequence of the elimination of proliferative cells and neural precursors from the cell population, which occurred during the purification of the terminally differentiated neurons (see Materials and Methods). It is also possible that reduced expression in NT2-N reveals a decrease or elimination of SOX14 expression in some cell types present in the population of the differentiated neurons. Accordingly, in the following investigation we proceeded by further analysis at the single-cell level.

### SOX14 is expressed in neurons and other differentiated derivatives of NT2/D1 and P19 cells

Based on the morphological criteria, NT2-N comprised phase-bright, neuron-like cells with small neurite outgrowths and small nuclei, which were growing on the top of phase-dark, large flat non-neuronal cells with large nuclei (Figure S1 B and C), as previously shown [29], [34], [39]–[41]. The morphological characteristics of the obtained cells were additionally visualized by α-Tubulin (Figure S1 E and F).

**Figure 2:**
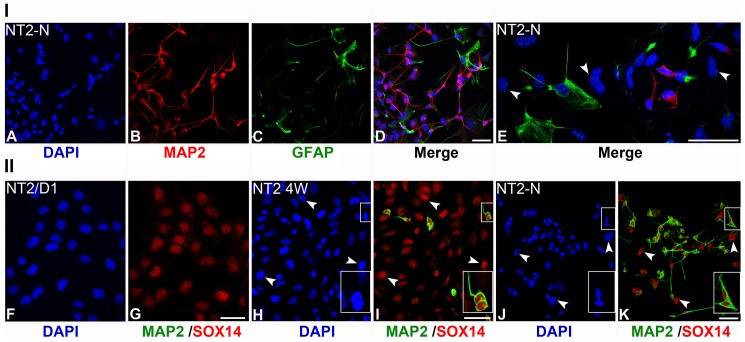
Immunocytochemical detection of MAP2, GFAP and SOX14 in NT2/D1, NT2 4W and NT2-N cells. **Panel I**: Immunocytochemical detection of MAP2 and GFAP-positive cells in NT2-N. **Panel II**: Immunocytochemical detection of MAP2 and SOX14-positive cells in NT2/D1, NT2 4W and NT2-N. The majority of cells in the NT2-N cell population are MAP2-positive neurons (B, D and E) with very few GFAP-positive astroglial cells (C, D and E). Cells with large nuclei that are immunonegative for both markers are designated by arrowheads in E. Specific SOX14 immunoreactivity/punctated nuclear signal was detected with different intensity in all undifferentiated NT2/D1 cells (F and G), in all MAP2-positive neurons in NT2 4W (H and I) and NT2-N (J and K), in non-neuronal cells in NT2 4W (arrowheads H and I), and NT2-N (arrowheads J and K). Boxed regions in H, I, J and K are enlarged in the same figures. Cell nuclei were counterstained with DAPI (A, D, E, F, H and J). Scale bars: 50 μm.

In order to confirm the presence of terminally differentiated neurons and astroglial cells, we performed immunostaining using specific antibodies for Microtubule-associated protein 2 (MAP2), a neuron-specific cytoskeletal protein implicated in determining and stabilizing dendritic shape during neuron development [42], and GFAP. The representative images of immunocytochemistry analysis (ICC) are presented in Figure 2, Panel I: A-E. Immunopositive cells were counted and a schematic representation of statistical analysis is given in Figure S2 A. Our result suggests that NT2-N consists of at least 3 different cell populations: MAP2+/GFAP-, MAP2-/GFAP+ and MAP2-/GFAP-. Nearly half of NT2-N cells (49%) were MAP2 positive, terminally differentiated neurons (Figure 2, Panel I: B, D and E). Among MAP2-negative cells we detected 4% GFAP-positive astroglial cells (Figure 2, Panel I: C, D and E), while 47% of large flat non-neuronal cells were shown to be GFAP-negative (arrowheads, Figure 2, Panel I: E).

Next we analyzed SOX14 expression in undifferentiated NT2/D1 cells, as well as in the populations of NT2 4W and NT2-N cells. The representative images of ICC are presented in Figure 2, Panel II: F–K. Immunopositive cells were counted and schematic representation of statistical analysis is given in Figure S2 B. We detected SOX14 expression as specific punctate nuclear staining in all undifferentiated NT2/D1 cells (Figure 2, Panel II: G). At the same time, those cells were MAP2 (Figure 2, Panel II: G) and GFAP immunonegative (data not shown).

Our results suggested that both NT2 4W and NT2-N consist of at least three different cell populations: MAP2+/SOX14+, MAP2-/SOX14- and MAP2-/SOX14+. In NT2 4W population, the majority (86%) were non-neuronal cells with large nuclei positive for SOX14 (MAP2-/SOX14+) (Figure 2, Panel II: H and I, arrowheads), while only 6% were neurons (MAP2+/SOX14+ cells) (Figure 2, Panel II: H and I). In comparison with NT2 4W, NT2-N population was enriched in MAP2+/SOX14+ neurons (50%), while percentage of MAP2-/SOX14+ decreased to 45% (Figure 2, Panel II: J and K). Interestingly, the level of SOX14 expression was lower in terminally differentiated MAP2+ neurons comparing to large flat non-neuronal cells (Figure S3). Only small percentage of cells in NT2 4W and in NT2-N (8% and 5% respectively) were negative for both markers. Accordingly, we concluded that the reduction of SOX14 expression in NT2-N population shown by Western blot (Figure 1B) is the result of the loss of non-neuronal cells with high SOX14 expression during purification step of the terminally differentiated neurons as well as of overall decrease of SOX14 expression in MAP2+ neurons.

**Figure 3:**
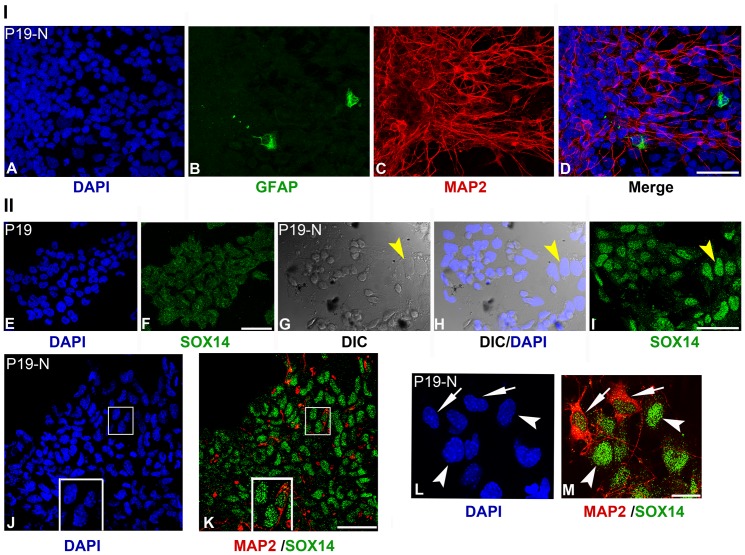
Immunocytochemical detection of MAP2, GFAP and SOX14 in undifferentiated P19 and differentiated P19-N cells. Panel I: Immunocytochemical detection of MAP2 and GFAP-positive cells in P19-N. Panel II: Immunocytochemical detection of MAP2 and SOX14-positive cells in P19 and P19-N. The P19-N population consists of a large number of MAP2 terminally differentiated neurons (C and D), and a few GFAP-positive astroglial cells (B and D). Specific SOX14 immunoreactivity/punctated nuclear signal was detected in a majority of cells in differentiated P19-N cultures (I, K and M), and at basal level in P19 cells (F). DIC transmitted light images show morphology of SOX14+ cells in P19-N population (G and H). Yellow arrowhead in G-I marks flat cells with large nuclei which show strong SOX14 immunoreactivity. SOX14 is expressed in MAP2-positive neurons (K, arrows in L and M) and in non-neuronal cells (K, arrowheads in L and M). Boxed regions in J and K are enlarged in the same figures. Cell nuclei were counterstained with DAPI (A, D, E, H, J and L). Scale bars: A–K 50 μm, L and M 20 μm.

Further, we analysed the presence of terminally differentiated neurons and astroglial cells in P19-N cells by applying immunostaining with MAP2 and GFAP-specific antibodies (Figure 3, Panel I: A-D), and schematic representation of statistical analyses of immunopositive cells is given in Figure S2 A. Analysis of attached EBs, differentiated for 7 days in culture (P19-N), revealed the presence of at least 3 different cell populations: MAP2+/GFAP-, MAP2-/GFAP+ and MAP2-/GFAP-. This population consisted of 36% MAP2-positive neurons and 1% GFAP-positive astroglial cells, while the remaining 63% were immunonegative for both markers (Figure 3, Panel I: A–D).

In accordance with Western blot results (Figure 1D), by ICC analysis we verified increased SOX14 expression in a population of P19-N (Figure 3, Panel II: I) compared to undifferentiated P19 cells (Figure 3, Panel II: F). We would like to emphasize that cells in P19-N population, which had high level of SOX14, correspond to flat cells with large nuclei (yellow arrowheads, Figure 3, Panel II: G–I). Additionally, double staining with antibodies specific for MAP2 and SOX14 in P19-N revealed the presence of at least 4 different cell populations: MAP2+/SOX14+, MAP2-/SOX14-, MAP2-/SOX14+ and MAP2+/SOX14- (Figure 3, Panel II: J–M; statistical analysis on Figure S2 B). In these populations 29% of MAP2-positive P19-N neurons were also SOX14 positive (Figure 3, Panel II: J–M; arrows on L and M) while strong immunoreactivity to SOX14 was observed in 45% of MAP2-negative, flat cells with large nuclei which were overlaid with MAP2+ cells (arrowheads, Figure 3, Panel II: L and M).

Comparative analysis of SOX14 expression at the single-cell level indicated that SOX14 expression was not consistent within populations of MAP2-positive neurons in NT2-N and P19-N cells. While all MAP2+ neurons in NT2-N were SOX14+, 5% of MAP2+ neurons were SOX14- in P19-N (Figure S2). Previous characterizations of NT2-N and P19-N differentiated derivatives have revealed heterogeneous sub-populations of neurons [40], [43]. It has been shown that dopaminergic, cholinergic, GABAergic and glutamatergic neurons were present within the NT2-N population [40], while the majority of P19-N represented GABAergic neurons [43]. It would be interesting to get further insight into the type of SOX14-positive neurons in populations of NT2-N and P19-N cells. Although the comparison between these two model systems indicated a significant similarity in the regulation of *SOX14/Sox14* gene expression, the presence of cell/species-dependent variations could not be excluded in comparative analysis.

Taken together, the data presented here revealed that *SOX14* expression is upregulated upon RA treatment in both human NT2/D1 and mouse P19 cell lines. The increased SOX14 protein level during *in vitro* neural differentiation indicated that both cell lines share similar molecular mechanisms underlying the regulation of *SOX14* gene expression. Moreover, by ICC we showed for the first time that both neuronal and non-neuronal cells obtained by *in vitro* RA-induced neural differentiation of NT2/D1 and P19 cells express SOX14.

### Expression of SOXB members during RA induced neural differentiation of EC cells

During neural differentiation, SOXB1 members suppress neurogenesis by maintaining neural cells in an undifferentiated state [12]–[13], and the balance between SOXB1 and *Sox21* expression determines whether neural progenitors remain in an undifferentiated state or begin the process of differentiation into neural cells [18]. Model presented by Sandberg et al. opens possibility that, *Sox14*, as the closest relative to *Sox21*, could have the similar role during neurogenesis.

**Figure 4:**
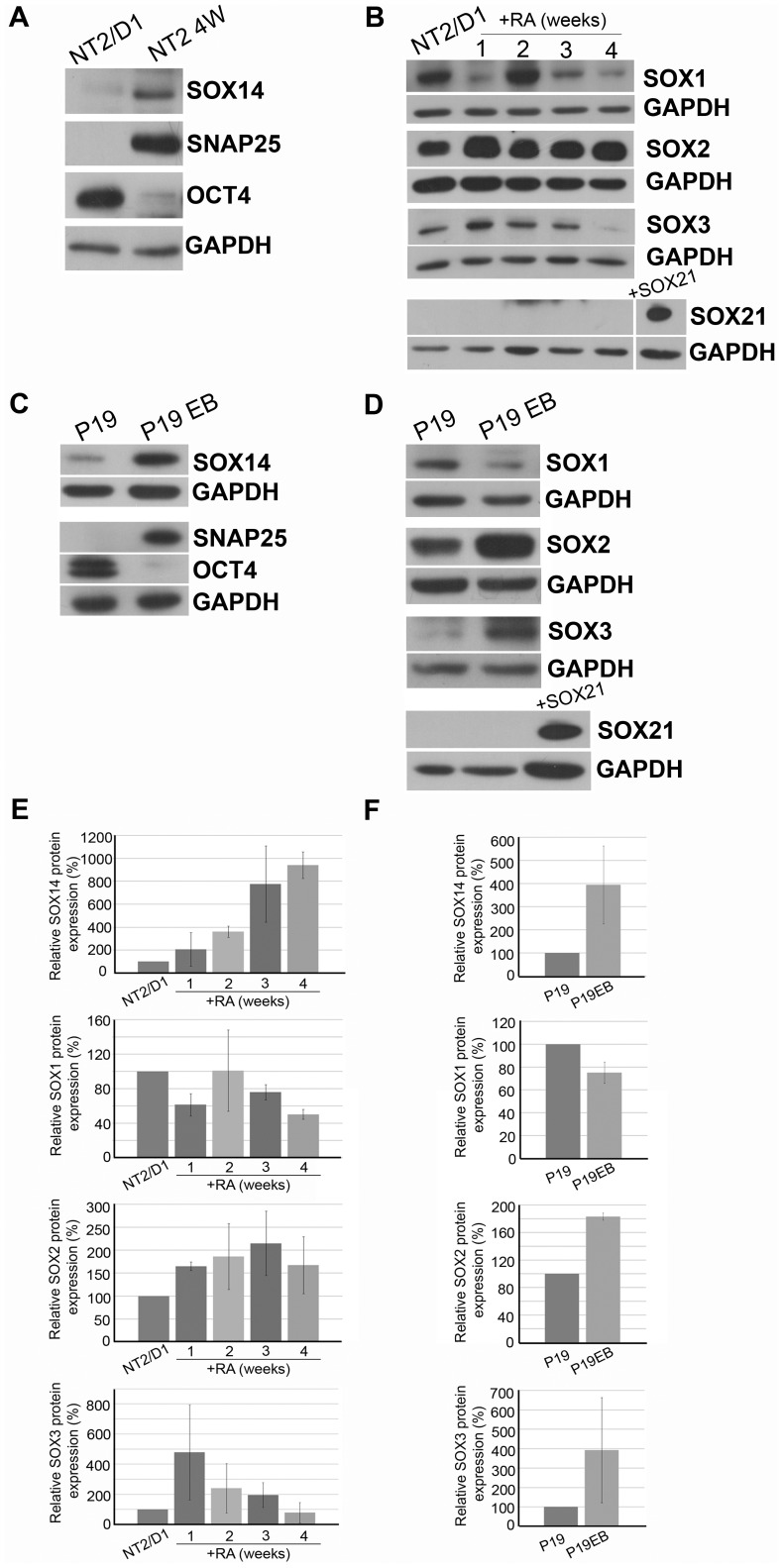
Expression of SOXB members during RA induced neural differentiation of EC cells. **A, C**: Western blot analysis of SOX14, SNAP25 and OCT4 expression in undifferentiated NT2/D1 and cells at final phase of RA induction (NT2 4W) and undifferentiated P19 and P19 cells at final phase of RA induction (P19 EB), respectively. **B**: Western blot analysis of SOX1, SOX2, SOX3 and SOX21 expression in NT2/D1 and cells treated with RA for 1, 2, 3 and 4 weeks. **D**: Western blot analysis of SOX1, SOX2, SOX3 and SOX21 expression in P19 and cells at final phase of RA induction (P19 EB). Protein extract from HeLa cells transiently transfected with SOX21 expression construct was used as a positive control for SOX21 expression. GAPDH was used as a loading control. Quantitative data of relative SOX14, SOX1, SOX2 and SOX3 protein levels during RA induction of NT2/D1 cells (**E**) and P19 (**F**) are summarized by the histogram. The quantities were calculated as a percentage of the quantity in control, untreated NT2/D1/P19 cells which were set as 100%. Data are presented as the means±SD of at least two independent differentiation experiments.

In order to compare the expression patterns of SOX14 with those of SOXB members during *in vitro* neural differentiation, we analyzed the overall protein expression of SOX1, SOX2, SOX3 and SOX21 at different time points during RA induction of NT2/D1, as well as at the final phase of the RA induction in P19 cells (P19 EB). The exit from pluripotency of NT2/D1 and P19 cells was confirmed by detection of diminished expression of the pluripotency marker OCT4 (Figure 4A and C) [44]–[45], while neural differentiation was confirmed by induction of the presynaptic plasma membrane protein SNAP25 (synaptosomal-associated protein 25) [46] at the final phase of RA induction (NT2 4W and P19 EB, Figure 4A and C, respectively).

As mentioned above, the expression of SOX14 increased during RA induction of human and mouse EC cells (Figures 1A and D, 4A and C). We demonstrated that all three members of SOXB1 subgroup were expressed in both model systems, but they exhibited distinctive expression profiles during neural differentiation. We detected fluctuation of the SOX1 protein level during NT2/D1 RA induction with tendency of decreasing at 3 and 4 weeks of treatmant (Figure 4B). SOX2 protein was gradually upregulated and its expression remained increased compared to untreated NT2/D1 cells (Figure 4B). The SOX3 expression was transiently upregulated after one week of RA induction, but then gradually downregulated up to 4 weeks of RA treatment (Figure 4B). However, the expression of SOX21 was not detected during RA induction of NT2/D1 cells in any of the analysed time points (Figure 4B). Relative quantification of SOX1, SOX2, SOX3 and SOX14 protein levels during RA induction of NT2/D1 cells is shown in Figure 4E.

By Western blot performed on P19 and P19 EB whole cell lysates we observed a downregulation of SOX1 and upregulation of SOX2 and SOX3 expression in P19 EB compared to untreated cells (Figure 4D). In line with the data obtained on NT2/D1 cells, no expression of SOX21 was detected in P19 cells (Figure 4D). Relative quantification of SOX1, SOX2, SOX3 and SOX14 protein levels in P19 and P19 EB cells is shown in Figure 4F.

Our data indicated that SOXB1 members are coexpressed with SOX14 during *in vitro* neural differentiation of human and mouse EC cells. The comparison of expression pattern of SOX14 with SOXB1 members in NT2/D1 revealed that the highest level of SOX14 expression is accompanied by downregulation of SOX1 and SOX3 and upregulation of SOX2 expression at 4 weeks of RA treatment. Also, increased SOX14 expression in P19 correlated with downregulation of SOX1 and upregulation of SOX2 and SOX3 at final phase of RA induction. Considering that the upregulation of SOX14 was accompanied by dynamic expression pattern of SOXB1 members during *in vitro* differentiation of NT2/D1 and P19 cells, we believe that this could indicate possible cross-talk between SOX14 and SOXB1.

Therefore, our next goal was to analyze if the ectopic expression of SOX14 could affect the expression of SOXB1 members in NT2/D1 and P19 EC cells.

### Ectopic expression of SOX14 in EC and HeLa cells

**Figure 5:**
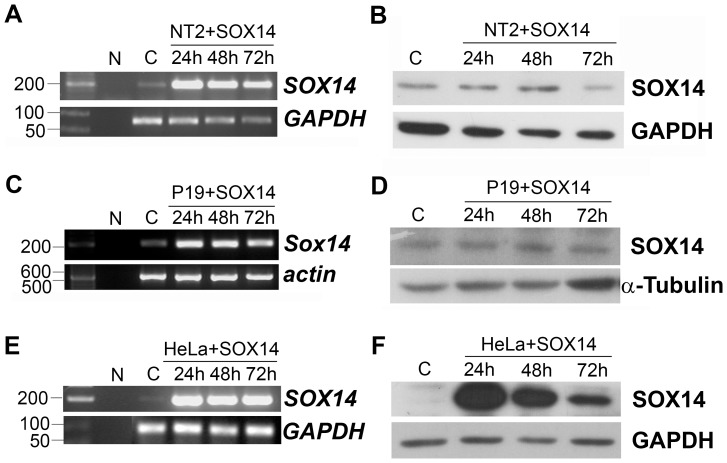
Ectopic expression of human SOX14 in NT2/D1, P19 and HeLa cells. **A, C, E**: Semi-quantitative RT-PCR on mRNA obtained from NT2/D1, P19 and HeLa cells, respectively, transiently transfected with pcDNA3.1/SOX14 expression construct. **B, D and F**: Western blot analyses on whole cell lysates obtained from NT2/D1, P19 and HeLa cells, respectively, transiently transfected with pcDNA3.1/SOX14 expression construct. SOX14 mRNA and protein levels were analyzed 24 h, 48 h, and 72 h after transfection. Transfection with pcDNA3.1 vector (designated as C) was used as a control for transfection. Negative PCR control is designated as N. GAPDH, α-Tubulin or *actin* were used as loading controls for Western blot and RT-PCR analyses.

We generated a *SOX14* expression construct by cloning its complete coding sequence into pcDNA3.1 expression vector, which was used in transient transfection experiments of EC cells. The ectopic expression of SOX14 was analyzed by semi-quantitative RT-PCR and Western blot of mRNA and whole cell lysates respectively obtained from NT2/D1 cells at 3 time points (24 h, 48 h and 72 h) after transfection. By semi-quantitative RT-PCR analysis we confirmed significant overexpression of *SOX14* at all tested time points, with a tendency to decrease between 48 and 72 h (Figure 5A). To our surprise, ectopic expression of SOX14 protein could not be detected in NT2/D1 cells at any of the tested time points, although various transfection agents and conditions were applied (Figure 5B). To test if this phenomenon is typical for SOX14 ectopic expression in NT2/D1 cells only, we performed transient transfection of these cells with the human *SOX3* expression construct generated in the same pcDNA3.1 expression vector. In contrast to the results obtained for SOX14, we successfully overexpressed SOX3 protein in NT2/D1 cells under the same experimental conditions (Figure S4). We also preformed transient transfection of pcDNA3.1/SOX14 expression construct in P19 cells. By semi-quantitative RT-PCR analysis we confirmed a significant increase in *SOX14* mRNA at the same time points (24 h, 48 h and 72 h) after transfection (Figure 5C), while Western blot analysis with SOX14 antibody failed to detect SOX14 protein overexpression in P19 cells as well (Figure 5D).

Taking into account that failure in translation of exogenous SOX14 could be a feature of pluripotent EC cells, we performed transient transfection of HeLa cells. In line with the data obtained in NT2/D1 and P19 cells, the *SOX14* mRNA level was significantly increased in HeLa cells 24 h, 48 h and 72 h after transfection (Figure 5E). On the other hand, by Western blot analysis we have confirmed the significant ectopic expression of SOX14 protein in HeLa cells, as presented in Figure 5F. For that reason HeLa cells were used in further functional analysis.

Inability to overexpress SOX14 protein in NT2/D1 and P19 cells might indicate that EC cells have a mechanism that keeps SOX14 protein at a particular level. We speculate that epigenetic mechanisms relying on miRNA might be involved in translation inhibition of *SOX14* mRNA in NT2/D1 and P19 cells. However, further work is needed to analyse molecular mechanisms involved in control of SOX14 protein level in EC cells.

### SOX14 acts as a transcriptional activator of a reporter gene in HeLa cells

SOX21, the closest relative of SOX14, has exhibited repression property on SOX2 in human glioma cells, as well as on Cdx2 in colon cancer and pluripotent stem cells [47]–[48]. While human SOX21 protein was proven to display repressor activity on target genes, trans-activation or repression properties of SOX14 transcription factor was not determined, mostly due to the lack of knowledge of its target genes.

**Figure 6:**
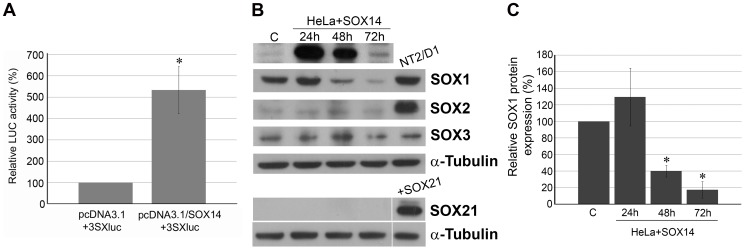
The effects of SOX14 ectopic expression. **A**: Effect of SOX14 ectopic expression on the activity of the SOX-responsive luciferase reporter gene. The plasmid 3SXluc was co-transfected into HeLa cells with either pcDNA3.1 vector or pcDNA3.1/SOX14 expression construct. Normalized luciferase activities were calculated as a fold of the 3xSX luc activity in cells co-transfected with vector pcDNA3.1+3SXluc, which was set as 100%. Data are presented as the mean ± S.E.M. of four independent transfections. Mean values of relative luciferase activities were compared with Student's t-test. The value of p≤0.01 is represented by *. **B**: Effects on SOX1, SOX2, SOX3 and SOX21 protein levels in HeLa cells analyzed by Western blot. Protein levels were analyzed 24 h, 48 h, and 72 h after transfection with pcDNA3.1/SOX14 expression construct. Transfection with pcDNA3.1 vector (designated as C) was used as a control for transfection. Protein extracts from NT2/D1 cells were used as positive controls for SOX1, SOX2 and SOX3 expression. Protein extract from HeLa cells transiently transfected with SOX21 expression construct was used as a positive control for SOX21 expression. α-Tubulin was used as a loading control. **C**: Quantification of the effects of SOX14 overexpression on SOX1 protein levels presented in B. The quantities of SOX1 protein in transfected cells were calculated as a percentage of the quantity in cells transfected with pcDNA3.1 vector, which was set as 100%. Data are presented as the means ± S.E.M. of three independent transfections experiments. Mean values were compared with Student's t-test. The value of p≤0.05 is represented by *.

In order to analyze the activation/repression property of human SOX14 protein, we performed co-transfection experiments in HeLa cells by studying the effect of SOX14 overexpression on the activity of SOX-responsive luciferase reporter gene (3SXluc) [36]. This reporter construct, which contains three SOX binding sites in front of the luciferase reporter gene, was already used for the analysis of SOX10 and SOX4 trans-activation properties [36]. As shown in Figure 6A, our data demonstrated that SOX14 overexpression increased luciferase reporter gene activity by approximately 5.5 fold. In contrast to previous literature data [7], our results revealed that human SOX14 acts as a transcriptional activator of SOX-responsive reporter gene in HeLa cells. By these data we showed for the first time that human SOX14 acts as a transcriptional activator of a responsive reporter gene in HeLa cells.

Accordingly, our results and literature data put into question the proposed division of SOXB genes on SOXB1 consisting of activators and SOXB2 comprising repressors. This division has already been challenged by the discovery of Kopp et al. demonstrating that upregulation of SOX2 represses *Nanog* and *Lefty1* expression [49]. SOX2 is not the only SOXB1 member with dual roles. It was also shown that SOX3 and Snail2 act as mutual transcriptional repressors in chicken embryos [50]. In addition, it was shown that SOX3, as a direct transcriptional repressor, inhibits expression of genes that are normally active in both the mesoderm and organizer of zebrafish embryos [51]. We may also speculate that lack of the proper genomic context present in the natural SOX14 target genes as well as the absence of proper co-factors in HeLa cells might influence the positive effect of SOX14 on the activity of the SOX-responsive reporter gene. However, resembling SOX2 activity, a dual role for SOX14 protein, acting as trans-activator as well as trans-repressor, could not be ruled out.

Taken together, we might suggest that activity of SOXB members is dependent on signalling networks, posttranslational modifications and cellular context, and that strict division of SOXB proteins into activators and repressors needs to be re-evaluated.

### SOX14 repressed SOX1 expression in HeLa cells

In addition to the functional link between SOXB1 and SOX21 shown during developmental of CNS [18], their cross-talk was also reported in cancer model system [47]. So far there is no experimental data providing support for such functional cross-talk between SOX14 and SOXB1 members. Accordingly, our next goal was to test whether SOX14 may affect SOXB1 members' expression in HeLa cells.

We studied the effect of ectopic SOX14 expression on SOX1, SOX2, SOX3 and SOX21. HeLa cells were transiently transfected with pcDNA3.1/SOX14 expression construct and whole cell lysates were isolated and analyzed by Western blot at 3 time points (24 h, 48 h and 72 h) after transfection (Figure 6B). Next, we analyzed the effect of SOX14 ectopic expression on SOX1, SOX2, SOX3 and SOX21 protein levels (Figure 6B). Contrary to previous reports [52]–[53] we detected SOX1 expression in HeLa cells, low levels of SOX2 and SOX3 expression and no expression of SOX21 (Figure 6B). Transfection experiments revealed no significant effects on SOX2, SOX3 and SOX21 expression (Figure 6B). Interestingly, our data demonstrated that SOX14 overexpression reduced SOX1 protein level (Figure 6B), which was quantified based on 3 independent transfection experiments (Figure 6C). The most prominent effect on SOX1 downregulation was obtained 72 h post-transfection (approximately a 5-fold decrease), while no significant effect was revealed 24 h post-transfection, when the highest level of ectopic SOX14 protein was observed (Figure 6B and C). The time needed for repression may suggest that SOX1 is not a direct SOX14 target gene and further work is needed to analyze molecular mechanisms underlying the role of SOX14 in downregulation of SOX1 expression. On the other hand, lack of an effect on SOX2 and SOX3 could be the consequence of model system used in this study. We cannot rule out the possibility that SOX2 and SOX3 could be affected by SOX14 in different model systems, in different cellular contexts.

By these data, for the first time, we have provided evidence for the functional link between SOX14 and a SOXB1 subgroup member. It is interesting to point out the opposite effect of SOX14 overexpression on SOX1, where it acts as a repressor, and on the SOX-responsive reporter gene, where it shows a trans-activating property in HeLa cells. As mentioned above, this is probably due to the lack of the proper genomic context in the reporter construct, which prevents SOX14 to act as a repressor. However, like other SOX family members, SOX14 may also have a dual role in the regulation of target gene expression, acting as either an activator or repressor, depending on cellular and genomic context.

It has been reported that SOX1 functions as a key regulator of neural cell fate determination and differentiation [54]–[56]. Apart from its role during neural differentiation, SOX1 is implicated in cancer development. It was shown that SOX1 is highly methylated in high grade squamous cell cervical carcinomas [49]. On the other hand, a genome-wide RNA interference (RNAi) screen in K-*ras* transformed NIH 3T3 cells identified that SOX14 is one of 28 genes required for Ras-mediated epigenetic silencing of the pro-apoptotic *Fas* gene [57]. Based on those findings, SOX14 could be involved in the positive or negative regulation of expression of genes implicated in epigenetic silencing, and its elevated expression could increase promoter hypermethylation of target genes, such as SOX1. Since SOX1 is considered as tumor suppressor [53], [58], the molecular mechanisms involved in the regulation of its expression, that rely on SOX14, gain additional significance. It would be interesting to explore how elevated expression of SOX14 influences HeLa cells' proliferation and invasiveness, and what impact decreased expression of SOX1 has on the aforementioned processes.

## Conclusions

In this paper we describe the expression pattern of SOX14 during *in vitro* neural differentiation of pluripotent human NT2/D1 and mouse P19 cells. We demonstrate that SOX14 expression is increased during RA induced neural differentiation of NT2/D1 and P19 cells, and it is detected in both terminally differentiated neuronal and non-neuronal cells. These data indicate that SOX14 is not exclusively a neuronal marker. The upregulation of SOX14 is accompanied by dynamic expression pattern of SOXB1 members during *in vitro* differentiation of NT2/D1 and P19 cells, which suggests that SOX14 could interfere with their expression. Accordingly, we analyzed the effect of SOX14 ectopic expression on protein levels of SOXB members. The overexpression of SOX14 protein is accomplished in HeLa cells only, so further experiments are performed in this model system. For the first time we demonstrate that ectopic SOX14 expression downregulates SOX1 in HeLa cells. The results obtained by transient transfection of HeLa cells are in correlation with expression pattern of SOX14 and SOX1 during the neural differentiation of EC cells. In particular, the upregulation of SOX14 expression observed at final phase of RA induction is accompanied by downregulation of SOX1 expression. Further experiments are needed in order to confirm their functional link during *in vitro* neural differentiation.

## Supporting Information

Figure S1 **Morphology of NT2/D1 and NT2-N cells following RA treatment.** The undifferentiated NT2/D1 cells grown in monolayer (A and D). Following RA treatment, NT2/D1 cells differentiate (NT2-N; B, C, E and F) into neuron-like cells (arrows in C, E and F) growing on the top of large flat cells with large nuclei (arrowheads in B, E and F). Cells were visualized by phase contrast (A–C) or by fluorescence following staining with α-Tubulin (D–F). Cell nuclei were counterstained with DAPI (blue color in D–F). Scale bar: (A–C) 20 μm, (D–F) 50 μm.
(TIF)

Figure S2 **Summary diagrams of statistical analyses of ICC results.** **A**: MAP2+/GFAP- cells, MAP2-/GFAP+ and MAP2-/GFAP- cells in NT2-N and in P19-N populations; **B**: MAP2+/SOX14+, MAP2-/SOX14+, MAP2-/SOX14-, MAP2+/SOX14- cells in populations of NT2 4W, NT2-N and P19-N. Percentages of cells presented in A and B were calculated against the number of DAPI-labeled cells. At least three separate fields of view were scrutinized with approximately 200 cells assessed.
(TIF)

Figure S3 **SOX14 expression on single cell level in NT2-N.** Specific SOX14 immunoreactivity/punctated nuclear signal was detected with higher intensity in cells with large nuclei that are immunonegative for MAP2 (designated by arrowheads in A, B, C and D) compared to MAP2+ neurons (designated by arrows in A, B, C and D). Scale bar: 20 μm.
(TIF)

Figure S4 **Overexpression of SOX3 protein in NT2/D1 cells.** NT2/D1 cells were transiently transfected with pcDNA3.1 vector or pcDNA3.1/SOX3 expression construct. Western blot analysis of SOX3 protein level was performed on cell lysates obtained 24 h post-transfection. Transfection with pcDNA3.1 vector (designated as C) was used as a control for transfection, while α-Tubulin was used as a loading control.
(TIF)
