# Supplementary figures and images for: Expression Analysis of SOX14 during Retinoic Acid Induced Neural Differentiation of Embryonal Carcinoma Cells and Assessment of the Effect of Its Ectopic Expression on SOXB Members in HeLa Cells
Source: PLoS One. 2014 Mar 17;9(3):e91852. doi: 10.1371/journal.pone.0091852 (PMC3956720; doi:10.1371/journal.pone.0091852)

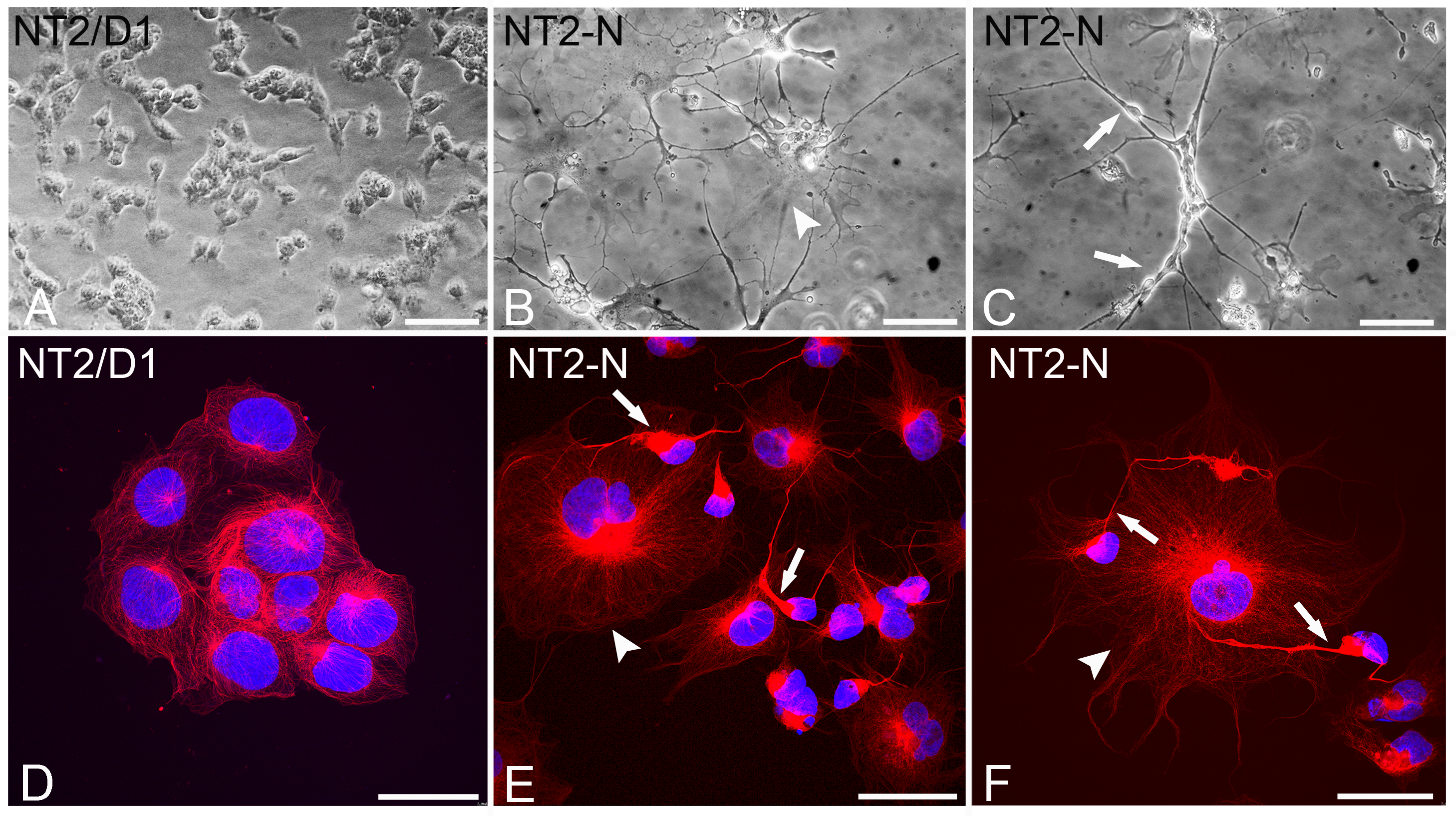

Supplement: Figure S1 — Morphology of NT2/D1 and NT2-N cells following RA treatment. The undifferentiated NT2/D1 cells grown in monolayer (A and D). Following RA treatment, NT2/D1 cells differentiate (NT2-N; B, C, E and F) into neuron-like cells (arrows in C, E and F) growing on the top of large flat cells with large nuclei (arrowheads in B, E and F). Cells were visualized by phase contrast (A–C) or by fluorescence following staining with α-Tubulin (D–F). Cell nuclei were counterstained with DAPI (blue color in D–F). Scale bar: (A–C) 20 μm, (D–F) 50 μm. (TIF) [file pone.0091852.s001.tif]

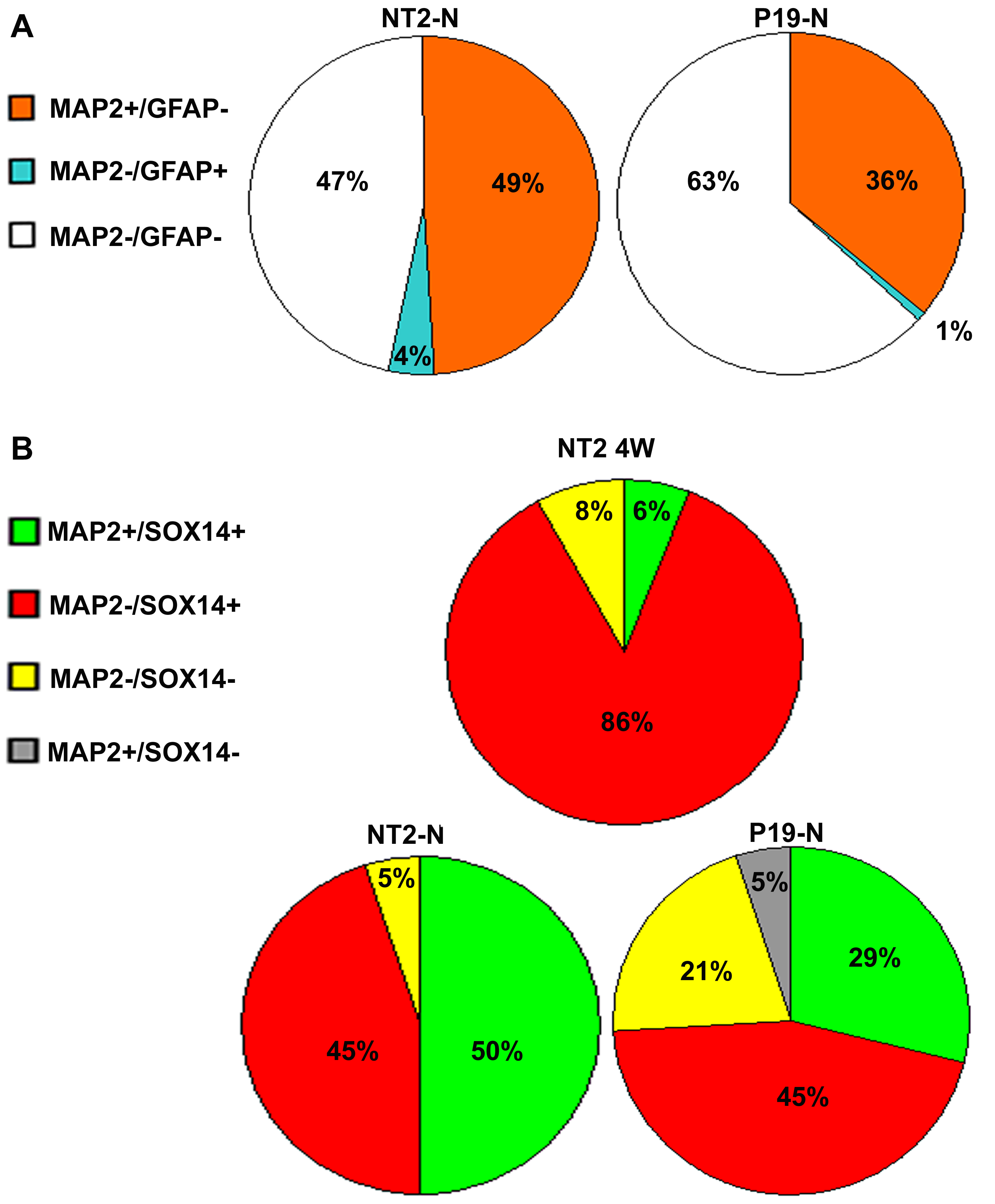

Supplement: Figure S2 — Summary diagrams of statistical analyses of ICC results. A: MAP2+/GFAP- cells, MAP2-/GFAP+ and MAP2-/GFAP- cells in NT2-N and in P19-N populations; B: MAP2+/SOX14+, MAP2-/SOX14+, MAP2-/SOX14-, MAP2+/SOX14- cells in populations of NT2 4W, NT2-N and P19-N. Percentages of cells presented in A and B were calculated against the number of DAPI-labeled cells. At least three separate fields of view were scrutinized with approximately 200 cells assessed. (TIF) [file pone.0091852.s002.tif]

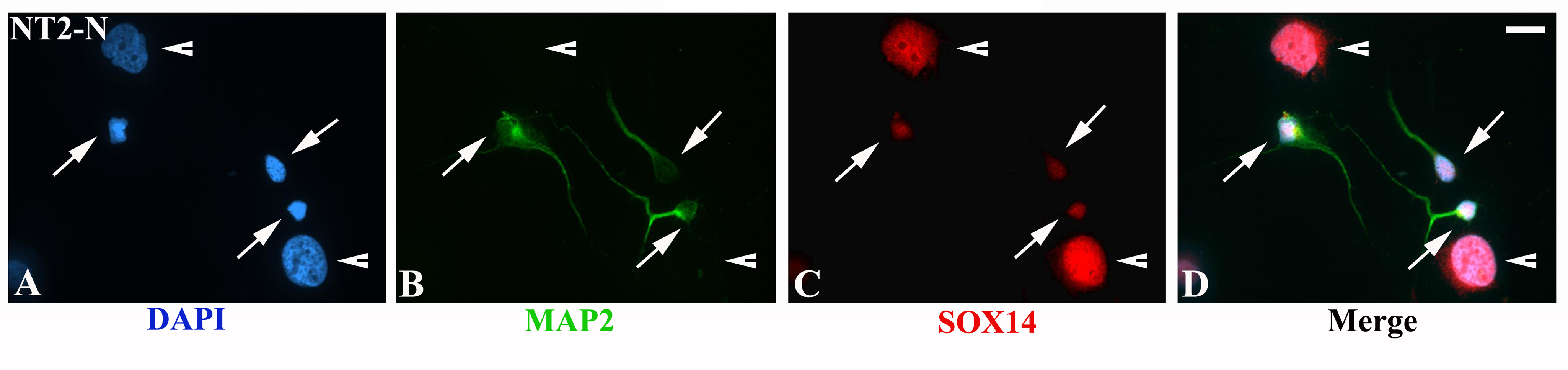

Supplement: Figure S3 — SOX14 expression on single cell level in NT2-N. Specific SOX14 immunoreactivity/punctated nuclear signal was detected with higher intensity in cells with large nuclei that are immunonegative for MAP2 (designated by arrowheads in A, B, C and D) compared to MAP2+ neurons (designated by arrows in A, B, C and D). Scale bar: 20 μm. (TIF) [file pone.0091852.s003.tif]

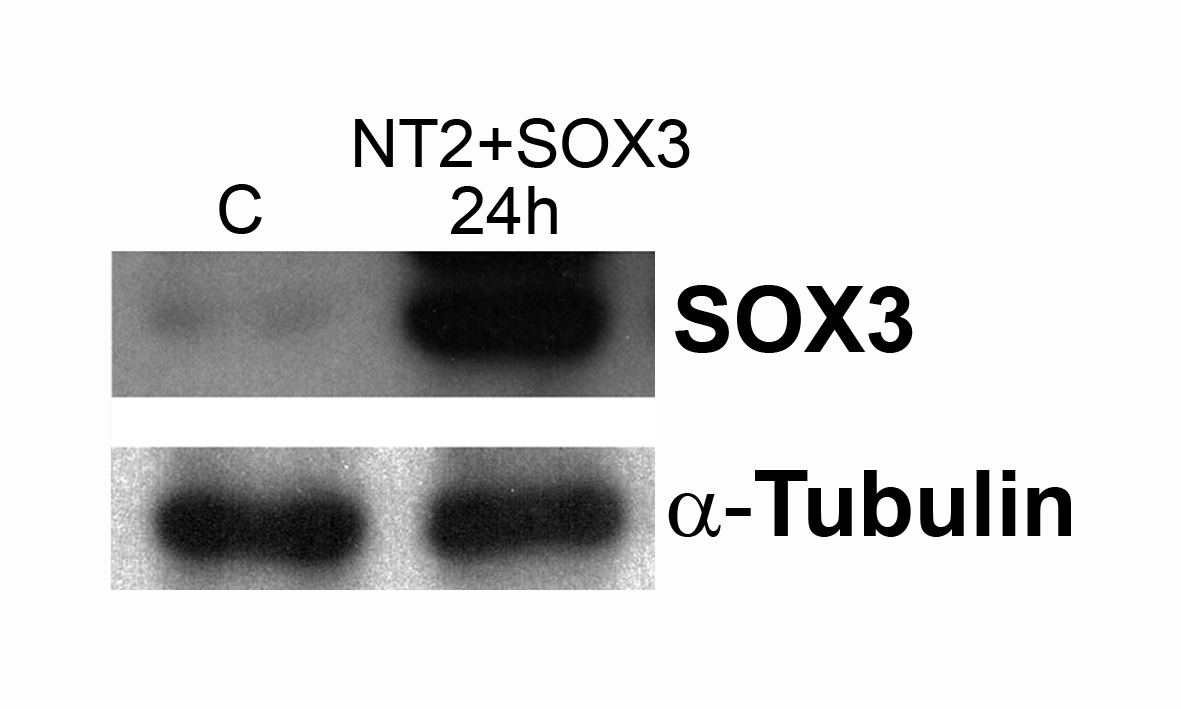

Supplement: Figure S4 — Overexpression of SOX3 protein in NT2/D1 cells. NT2/D1 cells were transiently transfected with pcDNA3.1 vector or pcDNA3.1/SOX3 expression construct. Western blot analysis of SOX3 protein level was performed on cell lysates obtained 24 h post-transfection. Transfection with pcDNA3.1 vector (designated as C) was used as a control for transfection, while α-Tubulin was used as a loading control. (TIF) [file pone.0091852.s004.tif]
